# Public Databases and Software for the Pathway Analysis of Cancer Genomes

**Published:** 2007-12-12

**Authors:** Ivy F.L. Tsui, Raj Chari, Timon P.H. Buys, Wan L. Lam

**Affiliations:** Cancer Genetics and Developmental Biology, British Columbia Cancer Research Centre, and Department of Pathology and Laboratory Medicine, University of British Columbia, Vancouver, Canada

## Abstract

The study of pathway disruption is key to understanding cancer biology. Advances in high throughput technologies have led to the rapid accumulation of genomic data. The explosion in available data has generated opportunities for investigation of concerted changes that disrupt biological functions, this in turns created a need for computational tools for pathway analysis. In this review, we discuss approaches to the analysis of genomic data and describe the publicly available resources for studying biological pathways.

## Background

**Figure 1.**
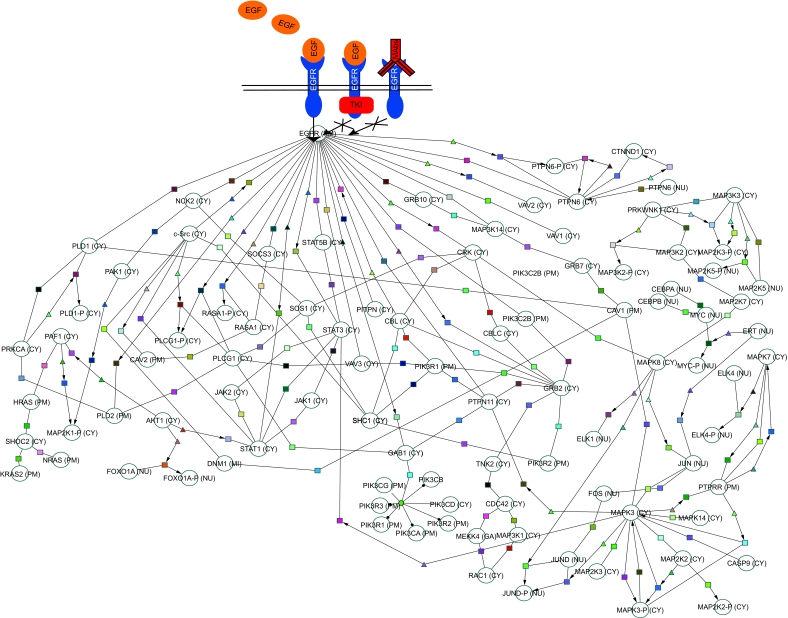
Example of EGFR-mediated signaling changes, a commonly disrupted pathway in lung cancer. The EGFR pathway could be disrupted by an increased expression of growth factor ligands. By targeting EGFR with tyrosine kinase inhibitors (TKIs) and MAb (monoclonal antibodies), EGFR activity can be eliminated. However, a downstream factor (e.g. MAPK signaling pathway) may also be activated to disrupt the pathway, thus making TKIs ineffective. Pathway data was obtained and selected from the Cancer Cell Map database and drawn using *Cytoscape*.

The development of cancer involves the accumulation of genetic and epigenetic alterations. Genetic events such as chromosomal rearrangements, changes in gene dosage, and sequence mutations can influence gene expression patterns, which contribute to the hallmark phenotypes of cancer^1,2^. The interaction between pathways and the involvement of pathways in multiple phenotypes complicate the interpretation of gene expression patterns. For example, the epidermal growth factor receptor (EGFR, HER1, ERBB-1) signaling pathway plays a role in specific phenotypes including resistance to apoptosis, increased proliferation, mitogenesis, transcription of numerous target genes, and actin reorganization, in several cancers (Fig. 1)^1,3,4^. In order to decipher the interaction within and between pathways, computational tools are necessary to annotate components, to identify co-regulated expression, and to identify sets of genes or pathways which are statistically over/under-represented within a dataset.

## Methods for Gene Classification

A major analytical step to mine large microarray data is sample classification or identification of gene sets with characteristic biological function. Entrez Gene at the National Center for Biotechnology Information (NCBI) provides unique identifiers for genes, and is a searchable database providing gene-specific information and links to external databases, including the Gene Ontology (GO) consortium, KEGG and Reactome^5^. A limitation of Entrez Gene is that genes are searched individually, which could be time consuming. Here, we describe the Gene Ontology (GO), a structural language to annotate gene functions for batch processing, and also methods of clustering analysis. The algorithmic basis of clustering identifies a pattern associated in a data set, which could be subsequently followed by GO analysis to identify its underlying biology.

### Gene Ontology annotation

The Gene Ontology (GO) Consortium was established in 2000 to provide a controlled vocabulary for annotating homologous gene and protein sequences in different organisms^6,7^. GO classifies genes and gene products based on three hierarchical structures that describe a given entry’s biological processes, cellular components, and molecular functions, and organizes them into a parent-child relationship^6^. Through easy on-line access (http://www.geneontology.org), the genome databases are being unified to expedite the process of retrieving information on genes and proteins based on shared biology among multiple organisms. Several software tools, including *GoMiner*^8,9^, *MAPPFinder*^10^, and *Onto-Express*^11,12^, have been developed to explore the GO relationships among high-throughput data. However, the biological functions of genes/proteins are often complex and annotating them into categories may oversimplify their biology. The flat-format output does not convey the richness of GO’s hierarchical structure. Nevertheless, this established system of nomenclature of genes and proteins is important for the interoperability of databases, batch processing, and the future design of pathway databases.

### Clustering

**Figure 2.**
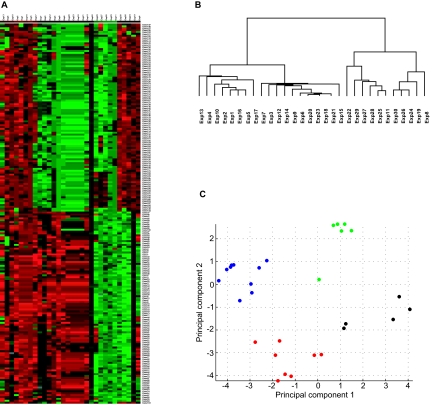
Graphical output display of heatmap, hierarchical clustering, and principal component analysis. **A:** An example of a heatmap representation of 30 simulated profiles helps the user to easily visualize four groups of samples along the x-axis with distinct characteristics expression patterns for 300 genes. Heatmap facilitates the grouping of altered genes and sample clusters, but does not convey any spatial relationship between clustered samples. **B:** An example of a dendrogram generated from hierarchical clustering of the simulated data represented in figure 2A. A dendrogram is a tree diagram consisting of many U-shaped lines connecting objects to represent hierarchical clusters. In this dendrogram, four clusters of samples are formed based on distinct expression signatures. **C:** A two-dimensional graphical visualization of principal components analysis (PCA) based on the simulated data shown in figure 2A. Samples are color-coded based on the four clusters observed by hierarchical clustering in 2B.

The biological system is integrative with tightly regulated processes, and genes with similar functions often exhibit coordinated expression patterns^13–16^. Transcriptional profiling studies typically aim to identify patterns of change among clinically related samples or to classify subgroups of samples^15–17^. Clustering of microarray data is widely used to identify groups of genes that display coordinated expression patterns performed in a supervised or unsupervised manner (Fig. 2)^13,14,17–21^. Unsupervised clustering is to classify data without *a priori* labeling of samples, whereas supervised clustering classifies data based on knowledge of samples type (e.g. cancer subtype)^21–24^. Clustering techniques are generally classified into two types: hierarchical and partitional^25,26^. Hierarchical clustering is constructed by either agglomerative (bottom-up) or divisive (top-down) approaches^25^. Agglomerative algorithms begin with separate clusters and merge them into successively larger clusters, while divisive algorithms begin with the whole dataset and divide the data into smaller clusters successively^25^. The output of agglomerative clustering is a tree of clusters called a dendrogram, in which each branch represents group of genes that have a higher order relationship (Fig. 2B)^25,27^. Partitional clustering directly reduces the dataset into a set of non-overlapping clusters^26^. Representative algorithms of partitional clustering include *k*-means clustering and self-organizing maps (SOM)^25^. *k*-means clustering requires the user to define *k* number of clusters^26,28^, and SOM partitions data into a two dimensional grid of clusters^13,29,30^. However, hierarchical clustering is more frequently used^17–20,30^. Detailed reviews of clustering algorithms are available and this topic will not be discussed further in this review^26,31–33^.

### Dimensionality reduction

Dimensionality reduction of data is used to minimize the number of input variables for finding coherent patterns of gene expression in an efficient manner^25,34,35^. Algorithms like principle component analysis (PCA) and multi-dimensional scaling (MDS) both employ this technique for classification procedures^25,34,36,37^. PCA visualizes multidimensional datasets by projecting data into a sub-space with 2 or 3 dimensions (Fig. 2C)^34,35,37,38^. The three-dimensional graphical display of MDS can be useful to portray relationships among the data points but might be complex to interpret and require subjective judgments.

Classification analysis may provide some pattern to the experimental datasets. Subsequently, the identified pattern may be further evaluated for biological interpretation using tools such as GO and/or Entrez Gene. However, the inherent limitation of pre-processed databases is subjective to the interpretation of the curator. Therefore, further validation should be considered. In a study that was conducted under the hypothesis that members in the same cluster would share related biological annotations, the majority of the clusters generated by three different clustering algorithms do not correspond well with known biology^39^. Furthermore, there is a need to improve the different clustering algorithms to enhance consistency of the results^39,40^. It is crucial to associate biological functions or regulatory pathways with each identified cluster of genes in order to deduce biological significance to each sample group^41^.

## Construction of Pathway Database

**Figure 3.**
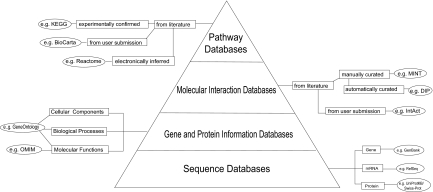
Biological knowledgebases contain a myriad of specific information on each gene/protein. Sequence databases are the basis for gene and protein information. Gene and protein information is further extracted and their inter-relationships are experimentally identified, building molecular interaction databases. All of this information is the foundation of pathway databases.

A remarkable number of published articles have collectively yielded thousands of molecular interactions for human and for model species. The challenge is to extract these individual interactions from the literature and to comprehend the dynamics of the interlocking networks as a whole. In recent years, massive efforts have been devoted to managing, integrating, and interpreting the available scientific information in a meaningful manner (i.e. building interactomes or networks of genes and pathways)^42,43^. Three categories of information are essential for the construction of interactome databases: gene and protein sequences, gene and protein biological information, and molecular interaction resources (Fig. 3). The major repositories of genes and protein sequences are listed in Table 1. Examples of nucleotide sequence databases include NCBI GenBank, EMBL, and DDBJ, all of which are part of the International Nucleotide Sequence Database Collaboration to facilitate data exchange and enhance accuracy^44–47^. The major databases for gene and protein biological information are listed in Table 2. Gene Ontology (GO), OMIM, Entrez Gene, and Universal Protein Resource Knowledgebase (UniProtKB) are the foundation for building these hierarchical databases^5,7,48,49^. The main publicly available molecular interaction databases are listed in Table 3. Currently, DIP, IntAct, MINT, HPRD, and MIPS all support the Human Proteome Organization (HUPO) Proteomics Standards Initiative Molecular Interaction (PSI-MI) standard format^50–55^. This is a unified data standard to represent molecular interaction data in a controlled vocabulary, which facilitates data comparison, exchange, and linking queries together^51^.

**Figure 4.**
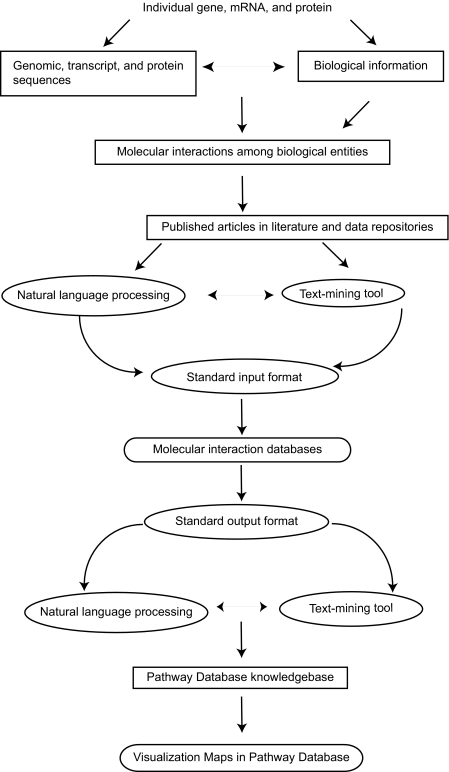
An approach to building pathway databases. Biological knowledgebases are represented as rectangles with squared edges. Computational tools for text-mining and language control are represented as ellipses. Molecular interaction and pathway databases are represented by rectangles with rounded edges.

The wealth of biological resources can complicate the construction of pathway databases (Fig. 4). When assembling information into a pathway database, developers must be cautious to distinguish those interactions that are deduced from hypothetical situations from those that have been experimentally confirmed. Within the latter group, care must also be taken to determine whether interactions have been confirmed in a single direct experiment or a high-throughput experiment. Furthermore, the use of natural language processing (NLP) systems to automate the extraction of information from published articles and to identify relationships between gene and protein names or interactions must be reviewed for biological relevance^56,57^. This method is useful as a first-pass tool for mining and extracting the knowledge in the literature. However, the constantly advancing nature of research, the further refinement of biological knowledge associated with each gene or protein further refining, the incompletion of the annotation database, and the complexity of entity names in the biological domain often makes it challenging for NLP to be high-quality with huge successes.

## Descriptions of Specific Pathway Database

Pathway databases facilitate the data mining process for cancer researchers. The major pathway databases are listed in Table 4. A collection of biological pathway and network databases is summarized in Table 5, including Pathguide: The Pathway Resource List (http://www.pathguide.org)^58^. This website is updated regularly and currently about 224 biological pathway resources are accessible through the Pathguide website. Here, we focus on a subset of databases that are publicly available.

### KEGG

The KEGG (Kyoto Encyclopedia of Genes and Genomes) database has been established since 1995 and has been one of the most popular knowledge databases to date^59^. The KEGG PATHWAY database consists of manually assembled pathway maps based on inspection of published literature. Pathway maps are grouped into metabolism, genetic information processing, environmental information processing, cellular processes, human diseases, and drug development. Most of the pathways associated with cancer are listed in the environmental information processing section, which is further subdivided into membrane transport, signal transduction, and signaling molecules and interaction. Beside human databases, information from other model organisms such as chimpanzee, mouse, rat, dogs, cows, and pigs is also available. KEGG pathway maps can be manipulated through the KEGG Markup Language (KGML) files, which provide graphical information to customize pathways.

### The Cancer Cell Map

The Cancer Cell Map (http://cancer.cellmap.org) is the only database that focuses on signaling pathways implicated in cancer. This resource contains ten cancer-related pathways and each pathway has approximately 100 to 400 interactions. Interactions are manually curated and reviewed for biological validity. Extensive information is provided in each pathway, including the cellular locations of the proteins, the types of physical interactions including molecular interaction, biochemical reaction, catalysis and transport, and post-translational protein modifications. The original citations, experimental evidences, and links to other databases are also listed. Gene expression data can also be visualized in the context of Cancer Cell Map pathways using the *Cytoscape* network visualization and analysis software^60^.

### Human Protein Reference Database

The HPRD (Human Protein Reference Database) contains ten cancer signaling pathways and ten immune signaling pathways which are graphically visualized in *GenMAPP* pathway maps^54,61^. The HPRD also offers the flexibility for investigators to refine their search of interested protein by multiple criteria, including molecular class from GO, domain name, motif, site of expression, length of protein sequence, molecular weight, and disease association (e.g. ovarian cancer and breast cancer). The protein domain architecture is graphically visualized with description of the domains and motifs within the queried protein. Post-translational modifications, protein interactions, and disease type are linked to PubMed, OMIM, Swiss Prot, Gene-Prot, Entrez Gene, or pathways within the HPRD. Individual genes within the pathway map are also linked to biologically relevant databases. Results from pathway analysis and HPRD entries can be readily exported. The use of XML (extensible markup language) for HPRD entries makes this database interoperable with other public databases. As with *Cytoscape*, the development of *GenMAPP* allows users to map microarray data onto pathway maps^61^.

### Reactome

Reactome is a publicly available, peer-reviewed resource of human biological pathways.^62^ Although the primary focus is on *H. sapiens*, it is now extending human pathways onto other organisms via putative orthologs to make them applicable to 21 model organisms, including mouse, rat, chicken, puffer fish, worm, fly, yeast, and *E. coli*. All the information in Reactome is cross-referenced with PubMed, GO, and the sequence databases at NCBI, Ensembl, and UniProt. Small molecules are linked to ChEBI (http://www.ebi.ac.uk/chebi), catalyst activities to the GO molecular function ontology, and sub-cellular locations to the GO cellular compartment ontology. The OMIM morbid map can be overlaid into the reaction map to see which genes have been implicated in the disease in the literature. Reactions from direct evidence in the literature and indirect evidence that are inferred via orthology in other species are indicated by color-coding. The *Reactome SkyPainter* tool facilitates the labeling of genes or proteins in the reaction maps. Thus, quantitative data from microarray experiments can be superimposed on Reactome maps to provide visualization and exploration in a pathway context.

## Visualization tools

Cross-talk between pathways can complicate the graphical representation of observed biological interactions. Therefore, visualization tools such as *Cytoscape*^60^ and *GenMAPP*^61^ have been developed to illustrate molecular interactions intuitively.

### Cytoscape

*Cytoscape* is a software tool for the integration of pathways with expression profiles. It allows the querying of networks by using several filtering tools, and linking a given network to public databases for functional annotations^60^. An important feature of *Cytoscape* is its extensible software framework which allows users to implement new algorithms and network computations. In addition to its use by the Cancer Cell Map (described above), *Cytoscape* can also be used in conjunction with other protein interaction databases or genetic interaction databases^63,64^. Molecular species are represented as nodes and intermolecular interactions are linked as edges. Different visual properties such as node color, shape, and size can be chosen, and subsets of nodes and edges can be displayed based on the criteria that are selected by the user. Visualization properties and analysis parameters are customizable.

### GenMAPP

*GenMAPP* (Gene Map Annotator and Pathway Profiler; previously called *Gene MicroArray Pathway Profiler*) is a computer program designed to display gene expression data in the context of biological pathways^61^. Based on the quantitative data that is loaded, the program will map genes onto relevant pathways and the user can set up criteria to color code the genes accordingly. *GenMAPP* visualize data in a file format called “MAPPs”, which allow users to organize the genes by their functional component. The user has the choice to download specific pathways or from the archive of MAPPs at www.netpath.org. The MAPPs database is manually curated, with interactions derived from textbooks, review articles, and public pathway databases. *Gen-MAPP* also has the feature to construct and modify the pathways by the user, a quality that is not possible if analyzing pre-existing pathway databases like EcoCyc, MetaCyc, and KEGG. Gene identification (ID) from GenBank, SWISS-PROT, Gene Ontology, or other known databases is used to link the gene object on the MAPP to public databases like SWISS-PROT or Entrez Gene by selecting the gene of interest. In addition, *GenMAPP* displays gene expression levels and provides statistical analysis based on the representation of altered genes in a given pathway MAPP.

## Software Tools to Analyze HTP Data

*GoMiner*^8,9^, *MAPPFinder*^10^, and *EASE*^65^ are software tools developed to correlate gene expression changes with GO terms to categorize the biological processes, cellular components, or molecular functions that are statistically affected. However, visualization of the pathway networks is challenging and complicated. Many software tools have been developed for microarray researchers to analyze large scale high-throughput data within the context of biological pathways, including the above mentioned *Cytoscape* and *GenMAPP*. Some of the most commonly used software tools are listed in Table 6. Here, we describe some of the freely available software tools that provide graphical representations of gene networks.

### Pathway Processor

*Pathway Processor* is designed to visualize whole genome microarray data in the framework of metabolic networks and provides statistical significance of the reliability of each differentially expressed gene^66^. This program displays data based on the information from the KEGG pathway database*. Pathway Processor* is implemented as two programs: *Pathway Analyzer* and *Expression Mapper. Pathway Analyzer* is the portion responsible for the statistical analysis of pathway significance, while *Expression Mapper* facilitates the visualization of this data on KEGG pathway maps.

### Whole Pathway Scope

*Whole Pathway Scope* (WPS) is a software tool to analyze high-throughput microarray experiments by referencing pathway or gene information from KEGG, BioCarta, and Gene Ontology^67^. The internal database also includes information from the Genetic Association Database and MedGene Database to allow users to rapidly identify disease-associated genes and highlight them inside their network diagram or select them for further network manipulation. One of the key features is the ability to view multiple experiments simultaneously and color-code the expression value with its *p*-value. In addition, this software allows users to customize their own metabolic pathway and gene groupings with the option of using statistical analysis.

### Pathway Explorer

*Pathway Explorer* is a web-based service available at https://pathwayexplorer.genome.tugraz.at to map expression profiles of genes onto pathway maps extracted from KEGG, BioCarta, and Gen-MAPP^68^. This web-based service reduces the local requirement for computational resources. It offers customizable analysis of the data by analyzing in a single or multiple pathways, and a right-tailed Fisher’s exact test and false discovery rate analysis were applied to determine the significance of the different pathways. Multiple experiments can also be displayed simultaneously on a single pathway with corresponding expression values. Data is linked to publicly available biological databases (e.g. the NCBI Entrez cross-database search, OMIM, KEGG pathways). The online accessibility of *PathwayExplorer* enables visualization of DNA or gene expression profiles within the context of biological pathways in a rapid manner.

## Future Considerations

**Figure 5.**
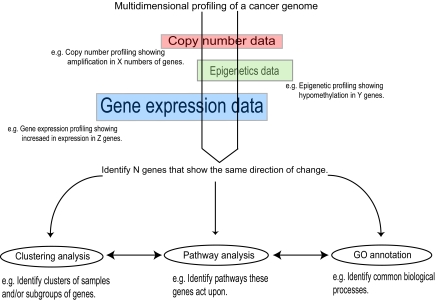
Genome-wide integrative analysis to identify pathways disrupted in cancer. Genome-wide analyses including copy number profiling, epigenetic profiling, and transcription profiling performed on the same cancer sample could narrow down the number of candidate genes, which would in turn help to pinpoint disrupted pathway involved in cancer.

The development of various computational tools to interrogate biological databases is accelerating the process to understand high-throughput genomic studies. However, these new tools pose new challenges, and one must be cautious about the limitations and errors associated with various databases. For example, it has been reported that when a partial Enzyme Commission (EC) number, which is a combination of four digits to annotate enzymatic activities without the fourth digit, is assigned to a gene, several pathway databases have used partial EC number annotations and inaccurately assigned them to a set of reactions that are associated with the same partial EC number under each orthology group^69^. Pathway database users should be aware of the possible inherent problems associated with any databases due to the variable quality of the published data. Comprehensive examination of the literature, as well as additional experimental validation, should be used to confirm any findings. Cross-platform integrative analysis of genomics, epigenomics, and transcriptional profiling will offer a deeper understanding of the biological complexity underlying disease processes (Fig. 5)^70^. The current challenge is to incorporate these data together for direct comparison, visualization, and analysis in order to identify salient gene candidates^71^. Once this is accomplished, the next step will be to place these candidates in the context of their proper signaling pathways for a given cancer type. Ultimately, the software programs used to do this should be intuitive to use, provide accurate information, allow customizable analyses, and offer sophisticated statistical tools. All of these features will be essential for characterization of disrupted gene networks in cancer. This will set the stage for rational therapeutic selection based on the underlying genetic realties of a specific tumor^38,41^.

## Figures and Tables

**Table 1.** Gene and protein databases.

**Database**	**Full name**	**Comments**	**Website**	**Ref**
NCBI Gen-Bank	NIH genetic sequence database	An international DNA sequence database	www.ncbi.nlm.nih.gov/Genbank	[45]
EMBL Nucleotide Sequence Database/EMBL-Bank	European Molecular Biology Laboratory Nucleotide Sequence Database	Collection of DNA and RNA sequences in Europe and is synchronized with GenBank at NCBI and DDBJ in Japan.	www.ebi.ac.uk/embl	[46]
DDBJ	DNA Data Bank of Japan	Nucleotide sequence database in Japan and in collaboration with EMBL and NCBI GenBank	www.ddbj.nig.ac.jp	[47]
Entrez Gene	-	NCBI database that focuses on gene-to-sequence relationship and provides gene-specific information.	www.ncbi.nlm.nih.gov/entrez/query.fcgi?db=gene	[5]
RefSeq	NCBI Reference Sequences	NCBI collection of non-redundant set of DNA, RNA, and protein sequences.	www.ncbi.nlm.nih.gov/RefSeq	[72]
UniGene	NCBI UniGene	Partitions GenBank sequences into sets of transcript sequences that are likely to represent distinct genes.	www.ncbi.nlm.nih.gov/entrez/query.fcgi?db=unigene	[73]
Ensembl	-	A source for comparative chordate genome sequences and gene annotation at EBI/Sanger.	www.ensembl.org	[74]
UCSC Genome Browser Database	University of California Santa Cruz Genome Browser Database	Human genome assembly and customizable track browsers at UCSC.	genome.ucsc.edu/bestlinks.html	[75]
UniProtKB/TrEMBL	Universal Protein Resource Knowledgebase/Translated European Molecular Biology Laboratories	Computer-curated protein sequence database containing translations of all coding sequences in EMBL/GenBank/DDBJ and also other protein sequences from the literature.	www.ebi.ac.uk/trembl	[49]
UniProtKB/Swiss-Prot Protein Knowledge-base	Universal Protein Resource Knowledgebase	Manually-curated protein sequence database providing publicly available information about protein sequences.	www.ebi.ac.uk/swis-sprot	[49]

**Table 2.** Gene and protein information databases.

**Database**	**Full name**	**Comments**	**Website**	**Ref**
GO	Gene Ontology	Provides a controlled vocabulary to describe gene and gene product attributes in many organisms.	www.geneontology.org	[7]
Entrez Gene	-	NCBI database that focuses on gene-to-sequence relationship and provides gene-specific information.	www.ncbi.nlm.nih.gov/entrez/query.fcgi?db=gene	[5]
OMIM	Online Mendelian Inheritance in Man	Collection of human genes information and genetic disorders.	www.ncbi.nlm.nih.gov/entrez/query.fcgi?db=OMIM	[48]
HomoloGene	-	Homolog detection among annotated genes of several eukaryotic genomes.	www.ncbi.nlm.nih.gov/entrez/query.fcgi?db=homologene	-
iHOP	Information Hyperlinked Over Proteins	Convert PubMed literature into a navigable resource.	www.ihop-net.org/UniPub/iHOP	[76]
SCOP	Structural Classification of Proteins	Classifies proteins of known structure based on their evolutionary and structural relationships.	scop.mrc-lmb.cam.ac.uk/scop	[77]
RCSB PDB	Research Collaboratory for Structural Bioinformatics Protein Data Bank	Resource for studying biomacromolecular structures and their relationships to sequence, function, and disease.	www.rcsb.org/pdb/Welcome.do;jsessionid=SvJzsMMl-0lENd1T-yXr7Q**	[78]
PIR	Protein Information Resource	A resource to identify and interpret protein sequence information.	pir.georgetown.edu	[79]
IntEnz	Integrated relational Enzyme database	Contains enzyme data curated and approved by the Nomenclature Committee of the International Union of Biochemistry and Molecular Biology	www.ebi.ac.uk/intenz	[80]
ENZYME nomenclature database	-	Database of information related to enzyme nomenclature.	ca.expasy.org/enzyme	[81]
BRENDA	BRaunschweig ENzyme DAtabase	Collection of enzyme functional data.	www.brenda.uni-koeln.de	[82]
Module Map	-	Collection and tools for the analysis of microarray data in 22 tumor types.	ai.stanford.edu/~erans/cancer	[83]
Cancer Gene Census	-	Catalogue of cancer-related genes.	www.sanger.ac.uk/genetics/CGP/Census	-
Cancer Gene Data Curation Project	-	Catalogue gene-disease and gene-drug relationships in cancer.	ncicb.nci.nih.gov/NCICB/projects/cgdcp	-
Tumor Gene Database	-	Database of tumor genes with a standard set of information.	www.tumor-gene.org/TGDB/tgdb.html	-
GEO	Gene Expression Omni-bus	A public archive for data submission and provides mining tools to query and download data.	www.ncbi.nlm.nih.gov/geo	[84]
CGED	Cancer Gene Expression Database	Database with graphical display of gene expression and clinical data on different tumor types.	cged.hgc.jp	[85]
Cancer Genes Resequencing Resource	-	Searchable database of cancer genes.	cbio.mskcc.org/cancergenes	-
SMD	Stanford Microarray Database	Database for storage and tools for processing and analyzing microarray data.	smd.stanford.edu/	[86]
Progenetix	-	A public database that collects information about chromosomal alterations in cancer.	http://www.progenetix.de/~pgscripts/progenetix/index.html	[87]
ArrayExpress	-	Public repository for microarray data.	http://www.ebi.ac.uk/microarray-as/aer/?#ae-main[0]	[88]
CGAP	Cancer Genome Anatomy Project	Database which relates chromosomal alterations to tumor characteristics.	http://cgap.nci.nih.gov/Chromosomes/Mitelman	[89]

**Table 3.** Molecular interaction database.

**Database**	**Full name**	**Comments**	**Visualization capability**	**Website**	**Ref**
IntAct	EBI protein intearction database	Protein interaction database by literature curation or user submissions.	HierarchView	www.ebi.ac.uk/intact/site/index.jsf	[52]
DIP	Database of Interacting Proteins	Curated both manually and automatically to combine experimentally determined protein-protein interactions.	Y	dip.doe-mbi.ucla.edu	[50]
MINT	Molecular INTeractions Database	Curated manually, experimentally verified protein interactions from literature.	MINT Viewer	mint.bio.uniroma2.it/mint/Welcome.do	[53]
HPRD	Human Protein Reference Database	Manually curated based on experimental evidence and contains information on domain architecture, post-translational modifications, interaction networks and disease association.	GenMAPP	www.hprd.org	[54]
HomoMINT	-	Molecular interactions discovered in model organisms are mapped to orthologs in Homo sapiens.	MINT Viewer	mint.bio.uniroma2.it/HomoMINT	[90]
Domino	Domain peptide interactions database	Protein interactions of domain peptides.	MINT Viewer	mint.bio.uniroma2.it/domino	[91]
PDZBase	-	Experimentally determined protein-protein interactions involving the PDZ-domains.	N	icb.med.cornell.edu/services/pdz/start	[92]
BOND	Biomolecular Object Network Databank	An interaction database that includes high-throughput data submissions and manually curated information from literature.	Cytoscape	bond.unleashedinformatics.com	[93]
BioGRID	General Repository for Interaction Datasets	A repository for protein and genetic interactions contributed by the community.	Osprey	www.thebiogrid.org	[94]
OPHID	Online Predicted Human Interaction Database	Database with known protein-protein interactions from human and predicted protein-protein interactions from model organisms.	Y	ophid.utoronto.ca/ophid	[95]
PIP	Potential Interactions of Proteins	Predicted protein-protein interactions derived from homology with experimentally known interactions from other species.	Y	bmm.cancerre-searchuk.org/~pip	[96]
MPPI	MIPS mammalian protein-protein interaction database	Published experimental protein interaction data in mammals	Y	mips.gsf.de/proj/ppi	[97]
HPID	Human Protein Interaction Database	Human protein interaction information and infer interactions between submitted proteins.	WebInter-Viewer	www.hpid.org or wilab.inha.ac.kr/hpid	[98]
InterDom	Database of Interacting Domains	Putative protein domain interactions information.	N	interdom.lit.org.sg	[99]
STRING	Search Tool for the Retrieval of Interacting Proteins	Database of known and predicted protein-protein interactions.	Y	string.embl.de	[100]

**Table 4.** Pathway databases.

**Pathway databases**	**Full name**	**Comments**	**Cost**	**Visualization capability**	**Website**	**Ref**
KEGG pathway	Kyoto Encyclopedia of Genes and Genomes Pathway	Manually drawn pathway maps with different organisms.	Free	Y	www.genome.ad.jp/kegg/kegg2.html	[59]
The Cancer Cell Map	-	Ten human cancer-related signaling pathways.	Free	Cytoscape	cancer.cellmap.org/cellmap	N/A
Reactome	-	Biological pathways that include experimentally confirmed, manually inferred, and electronically inferred reactions.	Free	Skypainter	www.reactome.org	[62]
HPRD	Human Protein Reference Database	Ten human cancer signaling pathways and 10 immune system signaling pathway.	Free	GenMAPP	www.hprd.org	[54]
BioCarta	Charting Pathways of Life	Graphical display of known and suggested pathways.	Free	Y	www.biocarta.com/genes/index.asp	N/A
STKE	Signal Transduction Knowledge Environment	Database of cellular signaling pathways.	Free	SVG	stke.sciencemag.org	[101]
PharmGKB	The Pharmacogenetics and Pharmacogenomics Knowledge Base	Database to explore relationships among drugs, diseases and genes, including their variations and gene products.	Free	Y	www.pharmgkb.org	[102]
Panther Classification System	Protein Analysis Through Evolutionary Relationships	Predict protein function and contains over 139 pathways mapped to protein sequences.	Free	CellDesigner	www.pantherdb.org/pathway	[103]
MetaCyc	Metabolic Encyclopedia of enzymes and pathways	Non-redundant, experimentally determined pathways from more than 900 different organisms.	Free	Y	metacyc.org	[104]
aMAZE	-	Molecular interactions and cellular processes.	Free	N	www.scmbb.ulb.ac.be/amaze	[105]
CGAP	Cancer Genome Anatomy Project	Pathways are from BioCarta and KEGG.	Free	Y	cgap.nci.nih.gov/Pathways	[106]
INOH Pathway Database	Integrating Network Objects with Hierarchies	Pathway database of different organisms which organize pathway objects in an ontology-based system.	Free	INOH Client tool	www.inoh.org	N/A

**Table 5.** Collection of databases.

**Databases**	**Full name**	**Comments**	**Cost**	**Website**	**Ref**
Pathguide	The Pathway Resource List	List about 222 biological pathway databases.	Free	www.pathguide.org	[58]
UBiC Bioinformatics Links Directory	UBC Bioinformatics Centre	Curated links to molecular resources, tools, and databases.	Free	bioinformatics.ubc.ca/resources/links_directory	[107]
NAR Molecular Biology Database Collection	Nucleic Acids Research online Molecular Biology Database Collection	Provide external links to sequence, structures, and pathway data-bases.	Free	www3.oup.co.uk/nar/database/subcat/6/25	[108]

**Table 6:** Software tools.

**Gene ontology (GO) analysis tools**	**Full name**	**Comments**	**Cost**	**Visualization**	**Knowledge-base**	**Website**	**Ref**
MAPPFinder	MicroArray Pathway Profiles Finder	View data in the context of Gene Ontology (GO) and GenMAPP biological pathways.	Free	Y	GO	www.genmapp.org/MAPPFinder.html	[10]
GoMiner	-	Tool to classify gene onto the Gene Ontology (GO) hierarchy framework.	Free	Y	GO	discover.nci.nih.gov/gominer	[8]
EASE	Expression Analysis Systematic Explorer	Statistical tool to analyze gene list by GO.	Free	Y	GO	david.abcc.ncifcrf.gov	[65]
Onto-Express	-	Analyze the list of genes into GO hierarchy.	Free	Y	GO	vortex.cs.wayne.edu/Projects.html#Onto-Express	[11]
GoSurfer	-	Analyze gene list using GO and visualize them as a hierarchical tree.	Free	Y	GO	bioinformatics.bioen.uiuc. edu/gosurfer	[109]
FatiGO	Fast Assignment and Transference of Information	Web-based tool to analyze and compare GO terms in 2 sets of gene list.	Free	Y	GO	fatigo.bioinfo.cipf.es	[110]

**Pathway analysis tools**	**Full name**	**Comments**	**Cost**	**Visualization**	**Knowledgebase**	**Website**	**Ref**
PathwayExplorer	-	Web-based tool to visualize data on publicly available biological pathways.	Free	KEGG, Biocarta, GenMAPP	KEGG, Biocarta, Gen-MAPP	pathwayexplorer.genome. tugraz.at	[68]
WholePathway-Scope	-	Pathway-based analysis tool to visualize BioCarta, KEGG, and GO term relationships.	Free	KEGG, Biocarta, GO	KEGG, Bio-carta, GO	www.abcc.ncifcrf.gov/wps/wps_index.php	[67]
PathwayExpress	-	Analyze the list of genes in the context of KEGG pathways.	Free	KEGG	KEGG	vortex.cs.wayne.edu/Projects.html#Onto-Express	[12]
Pathway Processor	-	Visualization and statistical analysis	Free	KEGG	KEGG	sysbio.harvard.edu/csb	[66]
Reactome Sky-painter	-	Tool to calculate statistical significance of affected pathways and visualize pathways.	Free	Skypainter maps	Reactome	www.reactome.org/cgi-bin/skypainter2?DB=gk_current	[62]
Oncomine	-	Cancer profiling database and provides web-based tools to analyze data.	Free	Scalable Vector Graphics (SVG)	KEGG, BioCarta, HRD, SOURCE	www.oncomine.org	[111]
DAVID	Database for Annotation, Visualization and Integrated Discovery	Offers various functional annotation tools to analyze gene list.	Free	DAVID Pathway Viewer	BioCarta, KEGG	david.abcc.ncifcrf.gov/home.jsp	[112]
GenePattern	A platform for integrative genomics	Tools for statistical analysis of data.	Free	heatmaps and other tools.	Multiple tools.	www.broad.mit.edu/cancer/software/genepattern	[113]
VisANT	Visualization and analysis tool for biological networks and pathways	A web-based application for the predicted functional links between genes and proteins analysis of biological networks and pathways.	Free	nodes and edges	Predictome, MIPS, BIND	visant.bu.edu	[114]
FatiGO+	Fast Assignment and Transference of Information	Compare distributions of GO or KEGG pathways between two groups of genes.	Free	KEGG	KEGG	babelomics.bioinfo.cipf. es/fatigoplus/cgi-bin/fatigoplus.cgi	[115]
PathwayStudio/PathwayAssist	-	Pathway analysis software.	License	Y	Manual curation	www.ariadnegenomics.com/products/pathway	[116]
Ingenuity Pathways Analysis	-	Pathway analysis software.	License	Y	Manual curation	www.ingenuity.com/products/pathways_analysis.html	N/A
MetaCore	-	Pathway analysis software.	License	Y	Manual curation	www.genego.com	N/A
PathArt	-	Pathway analysis software.	License	Y	Manual curation	jubilantbiosys.com/ppa.htm	N/A

**Visualization tools**	**Full name**	**Comments**	**Cost**	**Visualization**	**Knowledge-base**	**Website**	**Ref**
Cytoscape	-	Visualize molecular interactions and integrate gene expression profiles into pathways.	Free	Y	Retrieved from various databases.	www.cytoscape.org	[60]
GenMAPP	Gene Microarray Pathway Profiler	Visualize gene expression and genomic data on biological pathway maps.	Free	Y	Retrieved from various databases.	www.genmapp.org	[61]
Osprey	-	Graphical representation from Gene Ontology annotated interaction data by The GRID.		Y	The GRID	biodata.mshri.on.ca/osprey/servlet/Index	[117]
CellDesigner	-	Software to draw pathways.		Y	Panther Classification System	www.celldesigner.org	[118]
PathwayArchitect	-	Software for building pathways from databases or from own interaction data.	License	Y	-	www.stratagene.com/tradeshows/feature.aspx?fpId=90	N/A
